# Implications of the Presence of Hyperdense Middle Cerebral Artery Sign in Determining the Subtypes of Stroke Etiology

**DOI:** 10.1155/2021/6593541

**Published:** 2021-11-17

**Authors:** Suchada Sangpetch, Chayasak Wantaneeyawong, Atiwat Soontornpun, Nantaporn Tiyapun, Surat Tanprawate, Kitti Thiankhaw

**Affiliations:** ^1^Division of Neurology, Department of Internal Medicine, Faculty of Medicine, Chiang Mai University, Chiang Mai 50200, Thailand; ^2^The Northern Neuroscience Center, Faculty of Medicine, Chiang Mai University, Chiang Mai 50200, Thailand

## Abstract

**Background:**

Identifying stroke subtypes is crucial in choosing appropriate treatment, predicting outcomes, and managing recurrent stroke prevention.

**Objectives:**

To study the association of hyperdense middle cerebral artery sign (HMCAS) on noncontrast computed tomography (NCCT) brain and subtypes of stroke etiology.

**Methods:**

This is a retrospective hypothesis testing study. Patients aged 18 or over who had middle cerebral artery occlusion symptoms with HMCAS with verification on brain NCCT and received intravenous thrombolysis between January 2016 and June 2019 were enrolled. The demographic data, clinical outcomes, stroke subtypes, and characteristics of HMCAS were collected from medical records.

**Results:**

Ninety-nine out of 299 enrolled patients presented with HMCAS. The most common stroke subtype was cardioembolism (59%). Of the baseline characteristics, hypertension was more common in cases of large-artery atherosclerosis (LAA) (86.4%), and atrial fibrillation (AF) was the highest in cardioembolism (44.8%). HMCAS disappearance in cardioembolism was lowest compared to LAA and others (63% vs. 91% vs. 94.7%, respectively). The univariable analysis found that HMCAS disappearance is significantly associated with all stroke subtypes (Odds ratio, 95% confidence interval 10.58, 1.31-85.43; *P* = 0.027 for other and 5.88, 1.24-27.85; *P* = 0.026 for LAA). Multinomial logistic regression found that body weight and hypertension were associated with the LAA subtype. AF and intracranial hemorrhage (ICH) were associated with cardioembolism.

**Conclusion:**

The most likely diagnosis from the presence of HMCAS is cardioembolism, but the definite stroke etiologic subtype can not be identified. Combining the patient risk factors, including body weight, hypertension, and AF, with HMCAS and its characteristics will predict stroke subtypes more accurately.

## 1. Introduction

Stroke is the second leading cause of death in world populations older than 50 years (1). The global incidence of stroke is over 13.7 million each year, and the mortality rate from 1990 to 2016 is 86.52 per 100,000 population (2). Ischemic stroke is the most common type of stroke (3). The guidelines issued by the American Heart Association/American Stroke for the treatment of ischemic stroke recommend intravenous thrombolysis (IVT) for patients who come to the hospital within 4.5 hours after stroke onset (4). Patients who underwent IVT had 30% better clinical outcomes than those who did not receive IVT (5). The ischemic stroke subtypes can be classified into five groups to identify the etiology of stroke using TOAST (Trial of Org 10172 in Acute Stroke Treatment). Identifying the different subtypes will guide the physician in choosing an appropriate treatment, predicting outcomes and prognosis, and managing recurrent stroke prevention (6).

Hyperdense middle cerebral artery sign (HMCAS) is increased density of the middle cerebral artery (MCA) on brain noncontrast computed tomography (NCCT), indicating an acute intraluminal thrombus (7, 8). This is a CT sign of acute ischemia before any infarction becomes visible (9). HMCAS has a sensitivity of 27–54% and 100% specificity for MCA occlusion (10). HMCAS is found in 17-50% of patients with MCA territory stroke and is also associated with severe neurological deficits and poor functional outcomes (11, 12). As an intraluminal thrombus could result from embolism, arterial dissection, or atherothrombotic plaque (13), we hypothesize that the presence of HMCAS in NCCT might be associated with the prediction of a stroke subtype. In this study, we assess the factors associated with the presence of HMCAS so that we can analyze the relationship that can predict stroke subtype.

## 2. Material and Methods

We conducted a retrospective analysis of data from the stroke unit registry of Maharaj Nakorn Chiang Mai Hospital, the tertiary center of Chiang Mai University. These data were prospectively collected from 299 consecutive acute ischemic stroke patients aged 18 or over from January 2016 to June 2019 who received intravenous thrombolysis within a 4.5-hour onset of the symptoms. Patients diagnosed with acute ischemic stroke with middle cerebral artery occlusion symptoms and HMCAS from NCCT scan of the brain were enrolled. The demographic data (such as age, sex, and stroke risk factors) and clinical outcomes were collected from medical records. The subtypes of stroke etiology were classified following the TOAST classification, specifically large-artery atherosclerosis (LAA), cardioembolism, small-vessel occlusion (lacune), stroke of other determined etiology, and stroke of undetermined etiology (14).

NCCT images were produced using multidetector CT machines when patients visited the emergency department and were repeated 24 hours after IVT or in the case of clinical deterioration. The standard slice thickness was 1.5 mm. HMCAS was considered present when there was higher attenuation of MCA than in the adjacent segments of the artery. Clot location, disappearance after IVT, and type of clot disappearance were collected from the CT brain report interpreted by a board certificated diagnostic neuroradiologist using the picture achieving and communication (PAC) system. Two board-certified neurologists (KT and CW) were blinded to clinical data when assessing the mean clot length by CT planimetry using the radiologist's official CT report.

The National Institute of Health Stroke Scale (NIHSS) was used to quantify stroke severity and neurological outcome measurement at admission, 24 hours, and discharge. The functional outcome was evaluated using a modified Rankin scale (mRS) at admission and discharge. Neurological improvement was defined as an improvement in NIHSS at 24 hours of more than 4 points over baseline. The mRS at three months could not be obtained from the medical records; therefore, discharge mRS was substituted for evaluating a favorable functional outcome defined as mRS of 0-2. All forms of intracranial hemorrhage (ICH) and brain herniation in admission were reviewed as complications after IVT.

Descriptive analyses were used to describe the demographic data and characteristics of clinical features, outcomes, and HMCAS between the three groups. Continuous variables are displayed as mean with standard deviation or median with the interquartile range depending on data distribution. The analysis of variance (ANOVA) or Kruskal–Wallis test was used depending on the data to compare between the groups. Categorical data were analyzed using the chi-square or Fisher's exact test. Univariate logistic regression was performed to assess if the stroke subtype was associated with the outcomes and complications after IVT. Multinomial logistic regression was used to evaluate which factors could predict stroke subtypes. The *P* value for statistical significance was set at less than 0.05. All analysis was carried out using licensed Stata statistical software version 16.1.

## 3. Results

**Figure 1:**
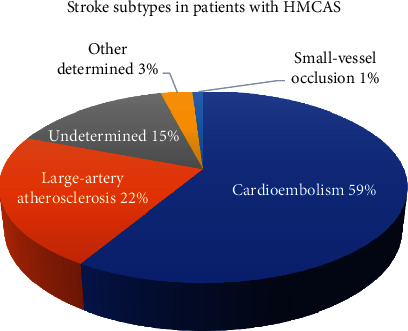
Stroke subtypes in patients with HMCAS.

We identified 299 patients who received IVT and were admitted to the acute stroke unit between January 2016 and June 2019. Ninety-nine patients presented with HMCAS. The subtypes of stroke etiology in these patients were identified using the TOAST classification, as shown in Figure 1. The most common subtype was cardioembolism, followed by LAA. The cause of the HMCAS in three patients in the other determined etiology subtype was carotid dissection. The patients with undetermined etiology included six patients who remained unknown as to cause despite extensive evaluation. Seven patients had more than one possible cause, such as having LAA with cardioembolic source, and two patients had suspected Embolic Stroke of Undetermined Source (ESUS).

The baseline characteristics of the enrolled patients grouped by stroke subtype are shown in Table 1. The mean age of the cardioembolic group is slightly higher than that of the LAA group but significantly higher than that of the other group (71.8 vs. 68.6 vs. 60.3 years, respectively, *P* = 0.016). Hypertension is commonly found in association with LAA (86.4%), followed by cardioembolism (63.8%) and others (26.3%). Atrial fibrillation (AF) is highest in cardioembolism (44.8%) but only found in 10.5% in the other group. There is no evidence of significant differences between subgroups regarding sex, body weight, hypercholesterolemia, diabetes, smoking, and prior stroke/transient ischemic attack (TIA). Baseline NIHSS is higher in cases of cardioembolism than in LAA groups (16.5 vs. 11.5 points, *P* = 0.021).

Clinical features and outcomes of the patients with HMCAS in different stroke subtypes are shown in Table 2. Among patients with HMCAS, newly diagnosed atrial fibrillation (NDAF) is discovered predominantly in cases of cardioembolism (39.7%), one patient in the other group and no one in the LAA group. ICH after IVT is significantly lower in the other group than in the cardioembolism and LAA groups (10.5 vs. 44.8 vs. 40.9%, respectively, *P* = 0.02). Disability of patients assessed by mRS at discharge showed that patients in the cardioembolic group tended to be more severe than those in the LAA group (4 vs. 3.5, *P* = 0.058). No significant differences in the rest of the outcomes are seen.

Characteristics of HMCAS in different stroke subtypes are shown in Table 3. HMCAS disappearance in cardioembolism is the lowest compared to the LAA group (63 vs. 91%). In all stroke subtypes, the most common thrombus location is seen in the M1 segment of MCA. HMCAS length and type of HMCAS disappearance show slight differences, but there is no statistical significance.

Univariable logistic regression in Table 4 was performed to evaluate the association between stroke subtype and the outcomes after IVT. HMCAS disappearance is statistically significant in association with all stroke subtypes (odds ratio (OR), 95% confidence interval (CI) other group 10.58, 1.31-85.43, *P* = 0.027; LAA 5.88, 1.24-27.85, *P* = 0.026). ICH is statistically significantly lower than the other subtypes (0.14, 0.03-0.68; *P* = 0.015).

Multinomial logistic regression was used to assess the factors that could predict stroke subtypes (Table 5). In this model, body weight and hypertension were associated with LAA subtype. The odds of LAA versus cardioembolism increased by 7% for a kilogram increase in body weight. Hypertension was around six times more associated with LAA than cardioembolism (OR, 95% CI 6.21, 1.32-29.15; *P* = 0.021). AF and ICH were associated with the cardioembolic subtype. The odds of LAA versus cardioembolism decreased by 97% in cases of AF compared to those without AF (OR, 95% CI 0.03, 0-0.30; *P* = 0.003). The odds of other versus cardioembolism decreased by 90% for those with ICH compared to those without ICH (0.10, 0.01-0.95; *P* = 0.045). HMCAS disappearance tends to be associated with the LAA and other subtype. The odds of the LAA versus cardioembolism was more than five times greater for those with HMCAS disappearance than those without (5.21, 0.82-33.12; *P* = 0.080); however, these results did not reach statistical significance.

## 4. Discussion

This study is aimed at finding any association between the presence of HMCAS in NCCT of the brain and stroke subtype. From our results, it is evident HMCAS can be found in every stroke subtype, but it was highest in cases of cardioembolism followed by LAA, undetermined, and other determined etiology (all are cases of arterial dissection). Kuo et al. (15) and Kim et al. (16) also found the same proportions. On the other hand, Niesten et al. (17) concluded that the presence of hyperdense vessel signs related to stroke subtype resulted from patients with cardioembolism, LAA, and dissection presenting with HMCAS at 45, 64, and 93%, respectively. The most likely diagnosis from the presence of HMCAS is the cardioembolic subtype, but the definite stroke etiologic subtype can not be identified.

The factors were then assessed to understand the relationships which can predict stroke subtype. We found that body weight and hypertension may be a predictor of LAA, while AF is a strong predictor of cardioembolism.

HMCAS disappearance represents vessel recanalization. In this study, HMCAS disappearance in LAA and other subtypes have a higher rate than in the cardioembolic subtype, unlike in the previous study of Forlivesi et al. (18), Puig et al. (19), and Molina et al. (20), which showed a lower rate of HMCAS disappearance in LAA. This difference might be due to the mean of the clot length in the cardioembolic group, which is relatively longer than in the LAA and the other group, even though it had no statistical significance. From the findings of Elofuke et al. (21), a shorter thrombus is more likely to disappear postthrombolysis, a finding supporting the higher rate of disappearance in the LAA and other groups. Overall, HMCAS disappearance rate in this study (75.8%) is higher than in the Khritonova et al. (22) study, which disappeared in about half of the cases and had outcomes twice as good as in persistent HMCAS patients. In those cases, IVT showed the benefits of recanalization in the patients who present with HMCAS.

Our study still found low neurological improvement and unfavorable functional outcomes. Over half of the patients with HMCAS had unfavorable outcomes of mRS, which were similar to the findings by Manelfe et al. (23), and there was no difference between subtypes. This might imply that stroke outcomes not only depend on the presence of recanalization but are also related to other factors such as time to recanalization or volume of the cerebral infarction. Further research may help in clarification of this finding. Zou et al. (24) concluded that HMCAS associated with hemorrhagic transformation had bleeding complications, 44.3% in the HMCAS group, compared to total ICH complications (37%) in our study. In comparison, the other group is the lowest ICH rate compared with LAA and the cardioembolic group. The explanation behind this finding may be due to the multiple etiology in the other group.

However, this study has some limitations. First, there is some missing data due to the retrospective nature of the study, so some variables were excluded. For example, we could not collect data pertinent to the clinically MCA infarction patients who did not have HMCAS, so we lacked a control group to evaluate the prevalence of HMCAS and outcomes between the Control and presence groups. Second, misclassification bias can occur because not all patients had a complete workup for stroke etiology, including CTA, echocardiography, and 24-hour Holter monitoring. The last one are some missing data of mRS at discharge. The correlation of HMCAS and early functional outcomes following IVT may not be demonstrated.

Even though this study cannot show a significant association between HMCAS and stroke subtype, the presence of HMCAS sheds more light on the condition so the physician can review the presence of cardioembolism first. However, clinical history, patient risk factors, and comprehensive investigations continue to be the main strategies in determining the definite stroke etiology. A further study on the prevalence and etiology of HMCAS is encouraged to provide early advanced management strategies such as mechanical thrombectomy and decrease poor clinical outcomes in these patients.

## 5. Conclusions

HMCAS is commonly found in acute ischemic stroke patients with cardioembolic etiology. However, it cannot be used as only one factor in identifying a specific stroke etiology. The clot characteristics, clinical outcomes, and patient risk factors provide greater detail in distinguishing cardioembolism from other etiologies. Therefore, determining the definite stroke etiologic subtype is based on adequate and sufficient evaluations of patient demographic data.

## Figures and Tables

**Table 1:** Baseline characteristics of patients.

Characteristics	Total (*N* = 99)	Cardioembolism (*n* = 58)	LAA (*n* = 22)	Others (*n* = 19)	*P* value
Age (years)—mean (±SD)	68 (15.2)	71.8 (14.1)	68.6 (13.3)	60.3 (18.1)	0.016
Male—no. (%)	52 (52.5)	27 (46.6)	14 (63.6)	11 (57.9)	0.343
Body weight (kg)—mean (±SD)	61.2 (14.8)	59.6 (14.8)	67.2 (13.2)	59.0 (15.2)	0.091
Risk factors					
Hypertension—no. (%)	61 (61.6)	37 (63.8)	19 (86.4)	5 (26.3)	<0.001
Hypercholesterolemia—no. (%)	42 (42.4)	27 (46.6)	10 (45.5)	5 (26.3)	0.294
Diabetes—no. (%)	21 (21.2)	14 (24.1)	6 (27.3)	1 (5.3)	0.145
Atrial fibrillation—no. (%)	29 (29.3)	26 (44.8)	1 (4.6)	2 (10.5)	<0.001
Smoker—no. (%)	46 (46.5)	23 (39.7)	14 (63.6)	9 (47.4)	0.158
Prior stroke or TIA—no. (%)	12 (12.1)	9 (15.5)	2 (9.1)	1 (5.3)	0.579
Thrombolysis					
Admission mRS—median (IQR)	4 (4-5)	4 (4-5)	4 (3-4)	4 (4-5)	0.215
ONT (minutes)—median (IQR)	141 (110-192)	140 (110-189)	152.5 (113-225)	124 (97-192)	0.528
DNT (minutes)—median (IQR)	52 (46-60)	52 (48-60)	48 (38-53)	57.5 (50.5-69.5)	0.008
ODT (minutes)—median (IQR)	87 (56-135)	87 (56-130)	105 (64-190)	74.5 (34-105.5)	0.117
Baseline SBP (mmHg)—median (IQR)	144 (130-167)	138 (124-170)	148 (132-167)	148 (134-162)	0.401
Baseline DBP (mmHg)—median (IQR)	81 (69-97)	80.5 (69-100)	86.5 (68-96)	81 (74-92)	0.987
Baseline NIHSS—median (IQR)	14 (8-20)	16.5 (10-21)	11.5 (7-13)	8 (8-17)	0.021
Baseline glucose (mg/dL)—median (IQR)	121 (102-155)	123.5 (105-155)	128 (103-215)	113 (96-140)	0.283
ASPECTS—median (IQR)	9 (8-10)	9 (8-10)	9 (9-10)	9 (8-10)	0.227

ASPECTS: Alberta Stroke Program Early CT Score; DBP: diastolic blood pressure; DNT: door-to-needle time; IQR: interquartile range; LAA: large-artery atherosclerosis; mRS: modified Rankin Scale; NIHSS: National Institute of Health Stroke Scale; ODT: onset-to-door time; ONT: onset-to-needle time; SBP: systolic blood pressure; SD: standard deviation; TIA: transient ischemic attack.

**Table 2:** Clinical features and outcomes in different stroke subtypes.

	Total (*N* = 99)	Cardioembolism (*n* = 58)	LAA (*n* = 22)	Others (*n* = 19)	*P* value
Clinical course					
NIHSS at 24 h—median (IQR)	9 (3-20)	13 (4-21)	5.5 (2-13)	7 (2-17)	0.126
NDAF—no. (%)	24 (24.2)	23 (39.7)	0 (0)	1 (5.26)	<0.001
Outcomes					
ICH—no. (%)	37 (37.4)	26 (44.8)	9 (40.9)	2 (10.5)	0.020
Brain herniation—no. (%)	22 (22.2)	17 (29.3)	3 (13.6)	2 (10.5)	0.163
NIHSS at discharge—median (IQR)	6 (2-15)	7.5 (2-18)	5 (2-11)	3 (1-9)	0.214
mRS at discharge—median (IQR)	4 (2-4)	4 (2-5)	3.5 (2-4)	3 (1-4)	0.058

ICH: intracerebral hemorrhage; IQR: interquartile range; LAA: large-artery atherosclerosis; mRS: modified Rankin Scale; NDAF: newly diagnosed atrial fibrillation; NIHSS: National Institute of Health Stroke Scale.

**Table 3:** Characteristics of HMCAS in different stroke subtypes.

Characteristics	Total (*N* = 99)	Cardioembolism (*n* = 58)	LAA (*n* = 22)	Others (*n* = 19)	*P* value
Thrombus location					
ICA—no. (%)	9 (9.1)	7 (12.1)	1 (4.6)	1 (5.3)	0.495
MCA-M1—no. (%)	59 (59.6)	30 (51.7)	15 (68.2)	14 (73.7)	
MCA-M2—no. (%)	31 (31.3)	21 (36.2)	6 (27.3)	4 (21.1)	
HMCAS length—median (IQR)	15.8 (11.8-22.8)	18.1 (12.2-24.2)	13.9 (11.9-21)	13.3 (9.4-25.2)	0.241
HMCAS disappearance—no. (%)	72 (75.8)	34 (63.0)	20 (91.0)	18 (94.7)	0.004
Type of HMCAS disappearance					
No longer seen—no. (%)	23 (31.9)	7 (20.6)	9 (45)	7 (38.9)	0.055
Decreased attenuation—no. (%)	42 (58.3)	25 (73.5)	10 (50)	7 (38.9)	
Migration—no. (%)	7 (9.7)	2 (5.9)	1 (5)	4 (22.2)	

HMCAS: hyperdense middle cerebral artery sign; ICA: internal carotid artery; IQR: interquartile range; LAA: large-artery atherosclerosis; MCA-M1: middle cerebral artery-M1 segment; MCA-M2: middle cerebral artery-M2 segment.

**Table 4:** Adjusted analysis: prognostic effect of stroke subtype on outcome measures.

Outcome measures	Cardioembolism			LAA			Other		
	No. (%)	OR (95% CI)	*P* value	No. (%)	OR (95% CI)	*P* value	No. (%)	OR (95% CI)	*P* value
HMCAS disappearance (*n* = 72)	34 (47.2)	1.00	—	20 (27.8)	5.88 (1.24-27.85)	0.026	18 (25)	10.58 (1.31-85.43)	0.027
Neurological improvement (*n* = 45)	28 (62.2)	1.00	—	10 (22.2)	0.89 (0.33-2.39)	0.822	7 (15.6)	0.63 (0.22-1.81)	0.387
Favorable functional outcome (*n* = 31)	16 (51.6)	1.00	—	6 (19.4)	0.98 (0.33-2.96)	0.978	9 (29)	2.36 (0.81-6.88)	0.115
ICH (*n* = 37)	26 (70.3)	1.00	—	9 (24.3)	0.85 (0.32-2.30)	0.753	2 (5.4)	0.14 (0.03-0.68)	0.015
Brain herniation (*n* = 22)	17 (77.3)	1.00	—	3 (13.6)	0.38 (0.10-1.46)	0.159	2 (9.1)	0.28 (0.06-1.36)	0.116

CI: confidence interval; HMCAS: hyperdense middle cerebral artery sign; ICH: intracerebral hemorrhage; LAA: large-artery atherosclerosis; OR: odds ratio.

**Table 5:** Multinomial logistic regression: factors which affect stroke subtype.

Factors	Multivariable analysis					
	LAA vs. cardioembolism		Other vs. cardioembolism		Other vs. LAA	
	OR (95% CI)	*P* value	OR (95% CI)	*P* value	OR (95% CI)	*P* value
Body weight	1.07 (1.02-1.14)	0.009	1.01 (0.96-1.06)	0.810	0.93 (0.87-1.00)	0.054
Hypertension	6.21 (1.32-29.15)	0.021	0.34 (0.07-1.62)	0.177	0.06 (0.01-0.39)	0.004
Atrial fibrillation	0.03 (0-0.30)	0.003	0.32 (0.05-2.00)	0.223	12.12 (0.68-215.37)	0.089
ODT	1.01 (1.00-1.02)	0.128	1.00 (0.98-1.00)	0.152	0.98 (0.96-1.00)	0.022
ICH	0.76 (0.19-3.02)	0.696	0.10 (0.01-0.95)	0.045	0.14 (0.01-1.58)	0.112
HMCAS disappearance	5.21 (0.82-33.12)	0.080	7.01 (0.74-66.63)	0.090	1.34 (0.08-22.43)	0.836

CI: confidence interval; HMCAS: hyperdense middle cerebral artery sign; ICH: intracerebral hemorrhage; LAA: large-artery atherosclerosis; OR: odds ratio; ODT: onset-to-door time.

## Data Availability

Data is available from the authors on request.

## Supplementary Materials

Supplementary Materials Supplementary files, including tables and figure, are available.
