# Supplementary material for: Implications of the Presence of Hyperdense Middle Cerebral Artery Sign in Determining the Subtypes of Stroke Etiology
Source: Stroke Res Treat. 2021 Nov 17;2021:6593541. doi: 10.1155/2021/6593541 (PMC8612777; doi:10.1155/2021/6593541)
Supplement: Supplementary Materials — Supplementary files, including tables and figure, are available. [file 6593541.f1.zip › Figure 1.docx]

**Figure 1. Stroke subtypes in patients with HMCAS.**
