# Supplementary material for: Implications of the Presence of Hyperdense Middle Cerebral Artery Sign in Determining the Subtypes of Stroke Etiology
Source: Stroke Res Treat. 2021 Nov 17;2021:6593541. doi: 10.1155/2021/6593541 (PMC8612777; doi:10.1155/2021/6593541)
Supplement: Supplementary Materials — Supplementary files, including tables and figure, are available. [file 6593541.f1.zip › Supplementary material ID 6593541 (Tables).docx]

**Table 1. Baseline characteristics of patients**

| **Characteristics** | **Total**  **(N=99)** | **Cardioembolism**  **(n=58)** | **LAA**  **(n=22)** | **Other**  **(n=19)** | ***P*-Value** |
| --- | --- | --- | --- | --- | --- |
| Age, years—mean (±SD) | 68 (15.2) | 71.8 (14.1) | 68.6 (13.3) | 60.3 (18.1) | 0.016 |
| Male —no. (%) | 52 (52.5) | 27 (46.6) | 14 (63.6) | 11 (57.9) | 0.343 |
| Body weight, kg—mean (±SD) | 61.2 (14.8) | 59.6 (14.8) | 67.2 (13.2) | 59.0 (15.2) | 0.091 |
| Risk factors | | | | | |
| Hypertension—no. (%) | 61 (61.6) | 37 (63.8) | 19 (86.4) | 5 (26.3) | <0.001 |
| Hypercholesterolemia—no. (%) | 42 (42.4) | 27 (46.6) | 10 (45.5) | 5 (26.3) | 0.294 |
| Diabetes—no. (%) | 21 (21.2) | 14 (24.1) | 6 (27.3) | 1 (5.3) | 0.145 |
| Atrial fibrillation—no. (%) | 29 (29.3) | 26 (44.8) | 1 (4.6) | 2 (10.5) | <0.001 |
| Smoker—no. (%) | 46 (46.5) | 23 (39.7) | 14 (63.6) | 9 (47.4) | 0.158 |
| Prior stroke or TIA—no. (%) | 12 (12.1) | 9 (15.5) | 2 (9.1) | 1 (5.3) | 0.579 |
| Thrombolysis | | | | | |
| Admission mRS—median (IQR) | 4 (4-5) | 4 (4-5) | 4 (3-4) | 4 (4-5) | 0.215 |
| ONT, minutes—median (IQR) | 141 (110-192) | 140 (110-189) | 152.5 (113-225) | 124 (97-192) | 0.528 |
| DNT, minutes—median (IQR) | 52 (46-60) | 52 (48-60) | 48 (38-53) | 57.5 (50.5-69.5) | 0.008 |
| ODT, minutes—median (IQR) | 87 (56-135) | 87 (56-130) | 105 (64-190) | 74.5 (34-105.5) | 0.117 |
| Baseline SBP, mmHg—median (IQR) | 144 (130-167) | 138 (124-170) | 148 (132-167) | 148 (134-162) | 0.401 |
| Baseline DBP, mmHg—median (IQR) | 81 (69-97) | 80.5 (69-100) | 86.5 (68-96) | 81 (74-92) | 0.987 |
| Baseline NIHSS—median (IQR) | 14 (8-20) | 16.5 (10-21) | 11.5 (7-13) | 8 (8-17) | 0.021 |
| Baseline glucose, mg/dL—median (IQR) | 121 (102-155) | 123.5 (105-155) | 128 (103-215) | 113 (96-140) | 0.283 |
| ASPECTS—median (IQR) | 9(8-10) | 9 (8-10) | 9 (9-10) | 9 (8-10) | 0.227 |

**Abbreviations:** ASPECTS, Alberta Stroke Program Early CT Score; DBP, diastolic blood pressure; DNT, door-to-needle time; IQR, interquartile range; LAA, Large-artery atherosclerosis; mRS, modified Rankin Scale; NIHSS, National Institute of Health Stroke Scale; ODT, onset-to-door time; ONT, onset-to-needle time; SBP, systolic blood pressure; SD, standard deviation; TIA, transient ischemic attack.

**Table 2. Clinical features and outcomes in different stroke subtypes**

|  | **Total**  **(N= 99)** | **Cardioembolism**  **(n=58)** | **LAA**  **(n=22)** | **Other**  **(n=19)** | ***P*-Value** |
| --- | --- | --- | --- | --- | --- |
| Clinical course | | | | | |
| NIHSS at 24 h—median (IQR) | 9 (3-20) | 13 (4-21) | 5.5 (2-13) | 7 (2-17) | 0.126 |
| NDAF—no. (%) | 24 (24.2) | 23 (39.7) | 0 (0) | 1 (5.26) | <0.001 |
| Outcomes | | | | | |
| ICH—no. (%) | 37 (37.4) | 26 (44.8) | 9 (40.9) | 2 (10.5) | 0.020 |
| Brain herniation—no. (%) | 22 (22.2) | 17 (29.3) | 3 (13.6) | 2 (10.5) | 0.163 |
| NIHSS at discharge—median (IQR) | 6 (2-15) | 7.5 (2-18) | 5 (2-11) | 3 (1-9) | 0.214 |
| mRS at discharge—median (IQR) | 4 (2-4) | 4 (2-5) | 3.5 (2-4) | 3 (1-4) | 0.058 |

**Abbreviations:** ICH, intracerebral hemorrhage; IQR, interquartile range; LAA, Large-artery atherosclerosis; mRS, modified Rankin Scale; NDAF, newly diagnosed atrial fibrillation; NIHSS, National Institute of Health Stroke Scale.

**Table 3.** **Characteristics of HMCAS in different stroke subtypes**

| **Characteristics** | **Total**  **(N= 99)** | **Cardioembolism**  **(n=58)** | **LAA**  **(n=22)** | **Other**  **(n=19)** | ***P*-Value** |
| --- | --- | --- | --- | --- | --- |
| Thrombus location | | | | | |
| ICA—no. (%) | 9 (9.1) | 7 (12.1) | 1 (4.6) | 1 (5.3) | 0.495 |
| MCA-M1—no. (%) | 59 (59.6) | 30 (51.7) | 15 (68.2) | 14 (73.7) |  |
| MCA-M2—no. (%) | 31 (31.3) | 21 (36.2) | 6 (27.3) | 4 (21.1) |  |
| HMCAS length—median (IQR) | 15.8 (11.8-22.8) | 18.1 (12.2-24.2) | 13.9 (11.9-21) | 13.3 (9.4-25.2) | 0.241 |
| HMCAS disappearance—no. (%) | 72 (75.8) | 34 (63.0) | 20 (91.0) | 18 (94.7) | 0.004 |
| Type of HMCAS disappearance | | | | | |
| No longer seen—no. (%) | 23 (31.9) | 7 (20.6) | 9 (45) | 7 (38.9) | 0.055 |
| Decreased attenuation—no. (%) | 42 (58.3) | 25 (73.5) | 10 (50) | 7 (38.9) |  |
| Migration—no. (%) | 7 (9.7) | 2 (5.9) | 1 (5) | 4 (22.2) |  |

**Abbreviations:** HMCAS, hyperdense middle cerebral artery sign; ICA, internal carotid artery; IQR, interquartile range; LAA, Large-artery atherosclerosis; MCA-M1, middle cerebral artery-M1 segment; MCA-M2, middle cerebral artery-M2 segment.

**Table 4. Adjusted analysis: prognostic effect of stroke subtype on outcome measures**

| **Outcome measures** | **Cardioembolism** | | | **LAA** | | | **Other** | | |
| --- | --- | --- | --- | --- | --- | --- | --- | --- | --- |
|  | **no. (%)** | **OR**  **(95% CI)** | ***P-*Value** | **no. (%)** | **OR**  **(95% CI)** | ***P-*Value** | **no. (%)** | **OR**  **(95% CI)** | ***P-*Value** |
| HMCAS disappearance (n=72) | 34 (47.2) | 1.00 | - | 20 (27.8) | 5.88 (1.24-27.85) | 0.026 | 18 (25) | 10.58 (1.31-85.43) | 0.027 |
| Neurological improvement (n=45) | 28 (62.2) | 1.00 | - | 10 (22.2) | 0.89 (0.33-2.39) | 0.822 | 7 (15.6) | 0.63 (0.22-1.81) | 0.387 |
| Favorable functional outcome (n=31) | 16 (51.6) | 1.00 | - | 6 (19.4) | 0.98 (0.33-2.96) | 0.978 | 9 (29) | 2.36 (0.81-6.88) | 0.115 |
| ICH (n=37) | 26 (70.3) | 1.00 | - | 9 (24.3) | 0.85 (0.32-2.30) | 0.753 | 2 (5.4) | 0.14 (0.03-0.68) | 0.015 |
| Brain herniation (n=22) | 17 (77.3) | 1.00 | - | 3 (13.6) | 0.38 (0.10-1.46) | 0.159 | 2 (9.1) | 0.28 (0.06-1.36) | 0.116 |

**Abbreviations:** CI, confidence interval; HMCAS, hyperdense middle cerebral artery sign; ICH, intracerebral hemorrhage; LAA, Large-artery atherosclerosis; OR, Odds ratio.

**Table 5. Multinomial logistic regression: factors which affect stroke subtype**

| **Factors** | **Multivariable analysis** | | | | | |
| --- | --- | --- | --- | --- | --- | --- |
|  | **LAA vs. Cardioembolism** | | **Other vs. Cardioembolism** | | **Other vs. LAA** | |
|  | **OR (95% CI)** | ***P-*value** | **OR (95% CI)** | ***P-*value** | **OR (95% CI)** | ***P-*value** |
| Body weight | 1.07 (1.02-1.14) | 0.009 | 1.01 (0.96-1.06) | 0.810 | 0.93 (0.87-1.00) | 0.054 |
| Hypertension | 6.21 (1.32-29.15) | 0.021 | 0.34 (0.07-1.62) | 0.177 | 0.06 (0.01-0.39) | 0.004 |
| Atrial fibrillation | 0.03 (0-0.30) | 0.003 | 0.32 (0.05-2.00) | 0.223 | 12.12 (0.68-215.37) | 0.089 |
| ODT | 1.01 (1.00-1.02) | 0.128 | 1.00 (0.98-1.00) | 0.152 | 0.98 (0.96-1.00) | 0.022 |
| ICH | 0.76 (0.19-3.02) | 0.696 | 0.10 (0.01-0.95) | 0.045 | 0.14 (0.01-1.58) | 0.112 |
| HMCAS disappearance | 5.21 (0.82-33.12) | 0.080 | 7.01 (0.74-66.63) | 0.090 | 1.34 (0.08-22.43) | 0.836 |

**Abbreviations:** CI, confidence interval; HMCAS, hyperdense middle cerebral artery sign; ICH, intracerebral hemorrhage; LAA, Large-artery atherosclerosis; OR, Odds ratio; ODT, onset-to-door time.
